# Genetic va riation in the na rrow-c lawed c ra yfish (Astacus lepto dactylus) po pulatio ns as assesse d by PCR-RFLP of mitochondria l *COI *gene

**Published:** 2015-12

**Authors:** Majidreza Khos hkholgh, Sajad Nazari

**Affiliations:** 1 Department of F isheries, Faculty of Natural Resources, University of Guilan, Sowmehsara, Iran; 2 Genetic and Breeding Research Center for Co ldwater F ishes, Yasouj, Iran

**Keywords:** *Astacus leptodactylus*, Mitochondrial DN A, PC R-RFLP, Genetic diversity

## Abstract

The genetic variation and population structure of narrow-clawed crayfish (*Astacus leptodactylus*) was examined by means of polymerase chain reaction (PC R) restriction fragment length polymorphism (RF LP) analysis of the cytochrome oxidase subunit I (*COI*) of mitochondrial DNA. A total of 194 adult specimens were collected from seven sample sites including, two in the south Caspian Sea and one each in Anzali wetland and Aras reservoir and three rivers Chafrood, Masule Rudkhan and S iah Darvishan. The PCR products were digested with 19 restriction enzymes and five enzymes revealed polymorphism patterns (*Dde*І, *Mbo*І, *Taq*I, *Rsa*І and *Hinf*І). Twenty eight composite haplotypes were showed with the number of haplotypes in each population sample ranging from 8 to 13. Private haplotypes were found at very low frequencies. Two regional (S iah Darvishan River and Astara) groups were clearly recognized by cluster and molecular variance model (AMOVA) analyses (*P*<0.0001). Each of these groups revealed dominant haplotypes while these haplotypes play less important rule in population structures of the other geographic areas. Intrapop ulatio n hap lo type (*h*) and nucleotide (*π*) diversities were high for each locality, ranging *h*=0.7560±0.030 and *π=*0.00334±0.00301, respectively. Results of this study discerned two divergent populations of narrow-clawed crayfish including S iah Darvishan River and Astara. Thus, the population structure of the narrow-clawed crayfish, as inferred from mtDN A analysis, is constituted by genetica lly separate groups that nearly reflect their geographic distribution.

## INTRODUCTION

The narrow-clawed crayfish *Astacus leptodactylus *is naturally and widely distributed in lakes, ponds and rivers throughout of Iran. *A. leptodactylus *is a widespread species and can be found throughout Europe, Eastern Russia, and the Middle East. However it is absent from some of the Northern European countries such as Norway and S weden, and the Southern European countries such as Spain and Portugal [1, 2]. The narrow-clawed crayfish is considered indigenous in the Eastern part of its range, but has been introduced into many of the Western European co untries [2, 3]. This species is found in both fresh and brackish waters, e.g. lagoons, estuaries, as well as running freshwater rivers in the Ponto-Caspian Basin [4, 5]. Iranian native crayﬁsh includes one crayﬁsh species, *Astacus leptodactylus*, with two subspecies, *A. l. leptodactylus *and *A. l. eichwaldi*. Only *A. leptodactylus *is commonly distributed in Iranian water resources. *A. leptodactylus *was found not only in the Caspian Sea up to 5–10 km offshore [6, 7]. The narrow-clawed crayfish is tolerant to changes in temperature, low oxygen content, and low water transparency, and is known to occur in saline conditions such as estuaries. Tolerance experiments indicated that juveniles and adults are well adapted for surviving salinities of at least 21ppt in the long term, and will tolerate being transferred directly back into freshwater. However, their ability to colonize the estuarine environment may be restricted to areas of low salinity (i.e. 7ppt) due to the adverse effects of seawater on egg development and hatching [4].

The advantages of mtDNA as a tool for population genetics have been extensively reviewed [8], as well as the ability of mtDNA to retain a history o f p ast isolation, even in the event of contemporary admixture of groups that evolved in allopatry [8-10]. Geographical investigations of mtDNA within several freshwater species have also demonstrated the significance of both historical biogeography and contemporary gene ﬂow in shaping intraspeciﬁc population genetic structure [11, 12]. F urther, mtDNA can aid in determining the taxonomic distinctiveness of individual populations and therefore assist in setting precedence for conservation programs [13]. Mitochondrial DNA has proved to be an superior tool for examining population genetics, above or below the species level [8, 14]. It has appeared as a genetic marker able to d istinguish stocks [15-20]. Because of the fast evolution and maternal mode of inheritance, mitochondrial DNA (mtDNA) has been widely used to survey genetic differences and evolutionary history between species and within species [8, 13, 21]. Variations of the complete mtDN A genome, individual genes, or restriction fragment length polymorphisms (RF LPs) have been applicable in characterizing taxa, establishing phylogenetic relationships, clarifying conspecific hybridizations, identification of hatchery and wild stocks and estimating stocks in many aquatic species [11, 13, 14, 19, 22, 23]. Many scientists have attempted to depict the genetic structure of freshwater crayfish species. A variety of methods have been applied. Early studies based on protein electrophoresis have shown only low levels of variatio n between populations of European freshwater crayfish [24, 25]. During the last decade molecular techniques with a higher degree of variability have been developed and applied successfully in population studies of freshwater crayfish. Genetic differentiation of populations had been assessed using mitochondrial DNA [17, 26-28], RAPD-PCR [29-31], AF LP [32] and microsatellites [31, 33-36]. P hylogenetic studies [37, 38], and reconstruction of the phylogeography [39] were possible using mitochondrial DNA. Regarding crayfish species, most of these studies focused on the *Austropotamobius pallipes *and information on genetic structure of *A. leptodactylus *is still rare. Assessing the relative levels of genetic diversity within and divergence among narrow-clawed populations in Iran can provide the first step towards conserving narrow-clawed genetic resources here and optimizing them for future breed improvement programs. This study is the ﬁrst molecular-based study of *A. leptodactylus *covering a large part of its distribution range, including river catchments of the south part of the Caspian Sea, Anzali lagoon, Southwest Caspian Sea and Aras reservoir in Northwest of Iran. The purpose of this study is to appraise the genetic status of the narrow-clawed crayfish breeds, by estimating both their relative levels of genetic diversity and the scope of genetic divergence among them using clustering procedure.

## MATERIALS AND METHODS

Adults of *A. leptodactylus *were collected from seven localities (Table 1, F ig. 1). From each locality between 14 and 37 individuals were collected and analyzed (Table 1). Tissues samples from walking legs (pereiopods) or abdomen were dissected on site, immediately fixed in 95% ethanol, and transferred to the laboratory.

The genomic DNA was isolated from each sample using standard phenol-chloroform extractions and ethanol precipitations following the method described previously [14,40]. Extracted DNA was verified for concentration using spectrophotometer (Nanodrop ND1000) and the DNA was following standardized to a specific concentration (i.e. 50 ng/ µl for PCR reactions and 100 ng/ µl for permanent storage in a DNA archive). The quality of each DNA specimen was approved visually on a 0.8% 0.5×TBE agarose gel containing ethidium bromide against a known standard.

The mtDN A cytochrome oxidase subunit I was amplified using the polymerase chain reaction (PCR). Primers were designed manually specifically for *A. leptodactylus *(alignment of primer sequences against the noble crayfish *A. astacus *accession number: I0DEU4 )*. *Primers 5′-GGT CAA CAA ATC ATA AAG ATA TTG-3′ (forward) and 5′- TAA ACT TC A GGG TGA CCA AAA AAT CA-3′ (reverse) were designed to amplify an approximate 1535 bp of the mtDNA *COI *gene. For amplification, the following reagents were added to each microtube: 2 µl of template DNA; 5 µl of 10× b uffer (100 mM Tris-HC l, pH 8.3, 15 mM MgC lR 2R , 500 mM KCl); 1 µl of each primer (10 pmol); 5µl of a 5 mM solution of each deoxyribonucleoside triphosphate (dNTP); 2.5 units of Taq DNA polymerase. Enough ultrapure water was added to each sample to make a solution of 50 µl. Polymerase chain reaction conditions consisted of 35 cycles of denaturation at 94°C for 1 min, annealing at 56°C for 1 min and extension at 72°C for 1 min (5 min for the last extension only). The amplicons were exposed to endonuclease digestion using the four-base recognitio n enzymes *Rsa *I, *Hinf *I and *Mbo*І and the five- base *Dde*I and *Taq*І. All restriction digestions followed the standard procedure provided by the manufacturer. Restriction digestion was carried out in a 10 µl volume containing 2 µl of PCR product, 2 units of restriction enzyme, 1 µl of the approp riate b uffer and 7µl of ultrapure water. RFLP digestion was performed in an incubator at 37°C for at least 16 h. Restriction fragments were segregated on a 6% acryl amid gel, stained with silver staining technique and photographed.

**Table 1:** Sa mpling localit ies , numbers , and nu mber of indiv iduals s ampled (*n*)

**Region**	**Map no.**	**S ampling site**	***n***
**Caspian Sea**	1	Astara	34
	2	Kiashahr	31
**Anzali lagoon**		Central p art	27
**River**	4	Chafrood	28
	5	M asuleh	14
	6	Siah d arvishan	23
**Reservoir**	7	Aras	37
**Total**			194

**Figure1:**
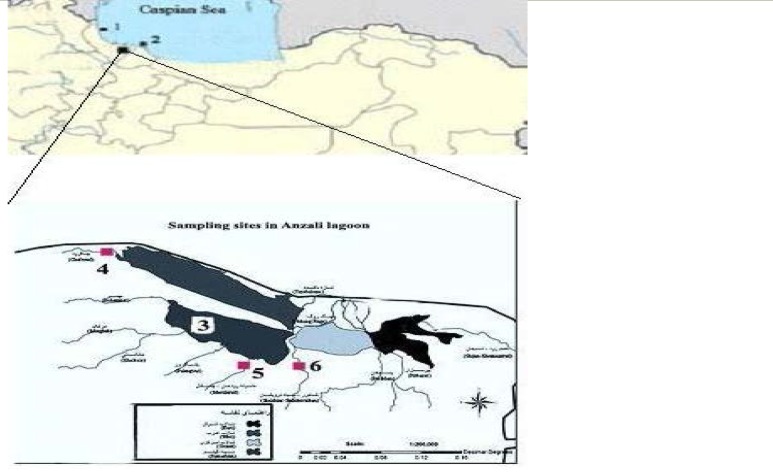
Sa mp ling s ites of the narrow-c lawed crayfis h, *A.* *leptodactylus*. The localities are labeled with numbers . Detailed informat ion about the s ites can be found in Table 1

A composite mtDN A haplotype, consisting of five letters that represent the fragment pattern generated by each of the restriction endonucleases, was compiled for each individual. The nucleotide d iversity (π) and haplotype diversity (*h *) in a population were computed according to known equation [41, 42]. The estimates of nucleotide divergence [42] between the mtDNA haplotypes and the samples examined were taken as standard genetic distances. The estimates were used for phylogenetic analysis performed with an unweighted pair group method with arithmetic mean (UPGMA) algorithm. C lustering robustness was calculated using bootstrap (100 iterations) [43,44]. Estimates of nucleotide divergence and dendrogram topology were made using the PAUP version 4.0b10 software package [45]. The main genetic variation indices were estimated using the REAP [46] and ARLEQUIN version 2.0 [28] software packages. Heterogeneity of haplotype frequencies between each sample pair was evaluated using the Monte Carlo method (1000 pseudorandom replicates [47]). Q uantitative estimates of the geographic subdivision of mtDNA variation were performed using the AMOVA method, where mo lecular variance was partitioned into three hierarchical levels, including the between- groups, between-population within groups, and among haplotype within population components [48, 49]. F-statistics criteria were calculated to test statistical significance of the hierarchic components of variance [50].

## RESULTS

Variation within samples in all 194 individuals amplified successfully in the mtDN A *COI *gene. Twenty eight different composite haplotypes were revealed with the number of haplotypes in each population sample ranging from 8 to 13 and 13 of them were private, that is, present in only one population sample (Table 2). Private haplotypes were found at very low frequencies, which were almost completely absent in other geographic areas (*P*<0.005).

**Table 2:** Nucleotide divers ity (π) and haplotype divers ity (*h*) values and geographic dis tribution of *A. leptodactylus* mtDNA RFLP co mpos ite haplotypes derived in this s tudy

	Ast ara (34)	Kiashahr (31)	Anzali (27)	Chafrood (28)		Masuleh (14)	Siah darvishan (23)	Aras (37)
	**π**	0.0035	0.0012	0.0017	0.0046	0.0032	0.0054	0.0036
	***h***	0.762	0.687	0.718	0.816	0.782	0.829	0.702
No. 1	Haplotypes AAAAA	5	11	8	7	3	4	12
2	AABAA	3	3	6	2	2	1	5
3	ABAAB	2	4	2	-	1	-	4
4	ABCAB	-	1	2	2	-	9	1
5	AABBC	13	3	1	-	3	-	1
6	ACBAB	-	-	-	-	-	-	2
7	BBBAC	1	-	-	-	-	-	-
8	BABCA	-	1	2	2	-	-	-
9	ACABD	-	-	-	-	-	-	1
10	ACCAA	3	4	-	1	-	-	-
11	BACBC	1	-	-	-	-	-	-
12	BACAC	-	-	-	2	-	1	-
13	ABBCD	-	-	-	-	-	1	-
14	ABAAD	-	-	1	1	-	-	1
15	ACACC	-	-	-	-	1	-	-
16	ABCAB	-	-	-	-	1	1	-
17	AACBD	-	-	-	-	-	1	-
18	ABABB	-	3	3	1	-	-	8
19	ABCAA	1	-	-	-	-	1	-
20	BAABA	3	-	1	2	2	-	-
21	BBACD	-	-	-	1	-	-	-
22	BBBAC	-	-	-	1	1	-	-
23	BAABD	-	-	-	-	-	-	1
24	BAABC	2	1	1	5	-	-	-
25	BACBC	-	-	-	-	-	1	-
26	BACCB	-	-	-	1	-	-	-
27	CCAAB	-	-	-	-	-	2	-

**Note: **Haplotypes are compos ite s cores for frag ment patterns produced by diges tion with *Rsa*І, *Hinf*І,*Dde*І, *Mbo*І and *Taq*I, res pectively. Nu mbers in parenthes es indicate numbers of individuals .

The geographic distributions of all haplotypes in throughout of sampling areas are given in Table 2. The most prevalent composite haplotype was haplotype 1 (AAAAA) with a frequency of 0.2577 in the all the samples. Frequencies of the non-private composite haplotypes varied among geographic areas. For example, haplotype 1 was found in all samples (Table 2). In the remaining geographic areas, it varied from 0.07 in Masuleh River to 0.32 in the Aras River. Haplotype 2 (AABAA) showed a frequency distribution with 0.11 in all geographic areas.

In the present study the mean haplotype diversity (*h *) and nucleotid e diversity (π) were 0.7560±0.030 and 0.00334±0.00301, respectively. The samples with the highest haplotype diversity were from S iah Darvishan River (0.829±0.046) and Chafroud River (0.816±0.032). On the other hand, the population samples with the lowest variability were from the southern part of the Caspian Sea in K iashahr with haplotype diversity0.687±0.025 as well as the Aras River (0.702±0.038) (Table 2). Table 3 demonstrates the results of nucleotide divergence methods to estimate the genetic differentiation of the populations examined. The estimation was based on the number of nucleotide substitutions. N ucleotide divergence ranged between 0.00149 and 0.01853 (Table 3).

**Table 3:** Pairwis e es timates of mt DNA d ivergence (% of nucleotide s ubs titutions ) in *A.* *leptodactylus*

**Locations**	**Astara**	**Kiashahr**	**Anzali**	**Chafrood**	**Masuleh**	**Siah darvishan**
**Kiashahr**	0.00614	-				
**Anzali**	0.00851	0.00149	-			
**Chafrood**	0.00615	0.00317	0.00268	-		
**Masuleh**	0.00178	0.00933	0.00832	0.00384	-	
**Siah**	0.01853	0.01624	0.00856	0.00981	0.01608	-
**Aras**	0.00727	0.00295	0.00382	0.00498	0.00694	0.01473

The lowest Nucleotide divergence was seen between K iashahr and Anzali samples. The results of the chi square test of *A.leptodactylus *populations test showed significant genetic structures in 8 of the 21 pairwise comparisons, most involving the S iah Darvishan River and Astara samples (Table 4). The χ2 test of haplotype frequencies also revealed that the population of S iah Darvishan River and the population of Astara are significantly different from one another (*P*<0.0001). It was shown that the main contribution to the heterogeneity of the population set was made by the *A. leptodactylus *samples from the S iah Darvishan River and Astara populations. Thus, three distinct genetic groups were identified (S iah Darvishan River Astara and all others). The cluster analysis also supported the existence of these three groups.

**Table 4:** Pairwis e comparis on of Monte Carlo bas ed chi-s quare values for *A.* *leptodactylus* populations fro m 7 locations (*P*-values in parenthes es ).

**Locations**	**Astara**	**Kiashahr**	**Anzali**	**Chafrood**	**Masuleh**	**Siah darvishan**
**Kiashahr**	58.32	-				
**Anzali**	89.66	22.12	-			
**Chafrood**	74.62	28.65	19.12	-		
**Masuleh**	98.54	16.03	22.09	11.78	-	
**Siah darvishan**	116.85	96.8	83.75	69.26	52.43	-
**Aras**	49.17	31.14	11.8	36.9	18.4	87.00

There were no clear subclustering and no correlation between geographic distribution and pairwise χ2 values between the two southern populations of Caspian Sea and other locations (F ig. 2). The most differentiated cluster included populations of the Astara and Siah Darvishan River populations. Aras and Anzali lagoon populations formed one population. Furthermore, the population positions on the dendrogram inferred from the mtDN A variation did not show clear differentiation among the *A. leptodactylus *populations from K iashahr and Anzali lagoon. Thus, the UP GMA tree suggested threedistinct groups; S iah darvishan River, Astara, Aras - Anzali lagoon- Chafrood River - Masuleh River (F ig. 2).

**Figure 2:**
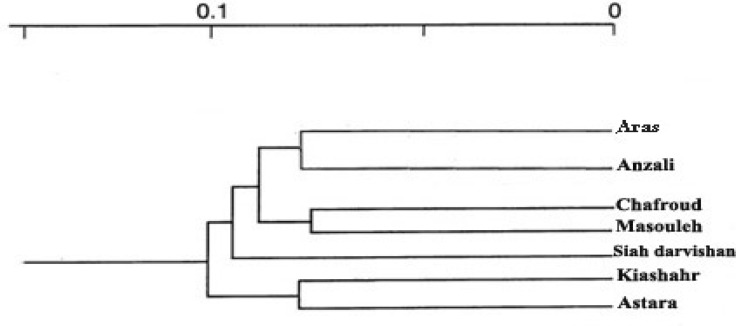
An UPGMA tree of the population genetic dis tances for the mtDNA *C* *OI* data fro m *A. leptodactylus*. Boots trap values are given at each node

The degree of genetic differentiation among all sample pairs was tested with analysis of molecular variance (AMOVA). The results of AMOVA demonstrated that collections between Astara in the southwest Caspian Sea and also Siah Darvishan River are significant (*P*<0.0001). Therefore heterogeneity test of the narrow-clawed crayfish populations for haplotype frequencies and Monte-Carlo with 1000 replicates in RFLP analysis of *COI *gene showed significant differences (*P*<0.0001) and these results showed that haplotype distribution in different location were significant and populations of S iah darvishan River and Astara statistically were significant (*P*<0.0001).

To make quantitative estimates of the values of genetic differences, total molecular

variance of haplo type frequencies was subdivided into three hierarchical levels (Table 5). An analysis showed that molecular divergence of *A. leptodactylus *was mostly distributed among haplotypes within populations (56.85%, Table 5). The AMOVA also segmented of total 38.79% genetic variation among the groups and had the low level of variance among populations within region (4.36%, *P*=0.143). However, according to the data acquired, the hierarchical levels of geographically isolated subdivisions were statistically significant (P<0.05). Thus concluded that the most part of the mtDNA intraspecific variation is determined by the differences among the haplotypes within a single population.

**Table 5:** Hierarch ic s earch for haplotype differences in *A. leptodactylus*

Source of v ariation	df	Percentage of variation	Fixation ind ices	*P*
Among group s	2	38.79	FR SCR =0.0228	<0.001
Among p op ulations within group s	4	4.36	FR CTR =0.0017	0.143
Among hap lotyp e within p op ulation	188	56.85	FR STR =0.0332	<0.001
Total	194			

## DISCUSSION

Results obtained from the analysis of mtDNA variation proved that most narrow- clawed crayfish samples display a wealth of diversity, presenting values of haplotype diversity. We found that the large mtDN A gene region (*C OI *gene) showed the high haplotype diversity. Detecting genetic variation in mtDNA between organisms depends chiefly on the evolutionary rate of genes and the number of nucleotide bases surveyed [11, 51]. As molecular variance of Astara and S iah Darvishan River are significant, two distinct populations were identified. O ur result is contrary to allozyme data which showed a very low level of genetic variability in several populations of *A. leptodactylus *[24] white-clawed crayﬁsh, *Austropotamobius pallipes pallipes *[52], noble crayﬁsh, *Astacus astacus *[25]. These studies mentioned that severe population size reduction due to several factors (such as overﬁshing, pollution, habitat destruction, etc.) tended to increase stochastic genetic effects leading to a loss of genetic variability within populations. UPGMA analysis built from haplotype frequencies clearly revealed a geographical structuring of populations, with two main clusters in *A. leptodactylus *corresponding to the southern part of Caspian Sea and S iah Darvishan River. This result suppo rts the hypothesis that each of these clusters include genetically differentiated populations. Most of the variation is distributed within populations, which may be explained by the existence of many shared haplotypes between populations. Analyses of the genetic structure of crustacean species with Caspian Sea tributaries distribution are relatively few and have mostly dealt with ﬁsh species [e.g. 15, 20, 23, 53]. In majority of the studies so far, a moderate to strong genetic cline between samp ling sites was observed, for example, Persian sturgeon [12, 14]. The geographical pattern of the genetic distribution, supported by the present data, is similar to those obtained in other freshwater organisms such as Persian sturgeon, *Acipenser persicus *[12, 19], ship sturgeon, *A. nudiventris*, [50], and Russian sturgeon *A. gueldenstaedtii *[54, 55].

This study, based on the *C OI *mtDNA marker, revealed a high number of coexisting haplotypes (28) in a restricted geographic range, indicating a strong genetic structure, with most localities containing just one or few ‘private’ haplotypes. Private haplotypes were, however, detected in low frequencies and therefore could not be used as population markers [14, 16, 26]. To discover the origin of the private haplotypes found within river catchments, additional populations nearby need to be examined.

The differences between stocks located close together were smaller than those between more distant stocks. However, it was not possible to determine whether low genetic variation found among individuals within a given stock was due to environmental constraints or genetic drift as result of a small population size. Nevertheless, the artiﬁcial translocation of individuals can be observed in the disjunct distribution o f some shared haplotypes between different drainage areas (F ig. 1) F ive of 7 populations showed no genetic diversity, while the most sample sites contained moderate level of genetic variability, especially because of the presence of private haplotypes. These levels of genetic variability are in accordance with the data of Grandjean and Souty-Grosset (2000) [26]. According to Avise et al., (1994) [2], contemporary intraspeciﬁc mtDNA variability is controlled by stochastic lineage extinction, which in turn is a function of long-term effective population size. Thus, the high genetic variability recorded within southern populations of Caspian Sea are not in accordance with ancient bottleneck events but suggested that these populations have had large effective populations sizes in the past [57, 58]. In the same manner, the genetic homogeneity revealed among Aras and Anzali lagoon populations suggests that these populations could be designated as one unique unit for conservation [11, 14, 33, 36, 39]. Several factors may be responsible for the observed pattern. For example, Overﬁshing, which increases the risks of effective population size reduction, and extinction of intermediate populations, may be one possibility. In addition, anthropogenic degradation of habitat could be an impediment to dispersal and final settling of larvae [1, 31, 33]. Data from the present study would be useful in assisting management and conservation, as well as notifying a recovery plan for wild populations. This is important specifically for a unique population such as Astara population, since the majority of the *A. leptodactylus *broodstocks for the hatcheries in Iran depend on wild populations [2, 59, 60]. Additional techniques such as microsatellite analysis and mtDN A sequences should be conducted to evaluate genetic variation within populations of narrow-clawed crayﬁsh to give particular attention to diverse populations and to more identify artificially stocked and naturally scattered populations.

## Notes

**Acknowledgments: **We thank the assistants A. Alavi and J. Ghasemzadeh in the Genetic Laboratory, the University of Guilan for their help in conducting the project. Financial support was provided, for the project by the Iran National Science Foundation (INSF), under the contract number 89004480.
